# The effects of mineral trioxide aggregate on osteo/odontogenic potential of mesenchymal stem cells: a comprehensive and systematic literature review

**DOI:** 10.1080/26415275.2020.1848432

**Published:** 2020-12-02

**Authors:** Danial Babaki, Sanam Yaghoubi, Maryam M. Matin

**Affiliations:** a Department of Biomedical Engineering, Tagliatela College of Engineering, University of New Haven, West Haven, CT, USA; b Visiting Scholar at Center for Cancer Research, National Cancer Institute, NIH, Bethesda, MD, USA; c Department of Biology, Faculty of Science, Ferdowsi University of Mashhad, Mashhad, Iran; d Novel Diagnostics and Therapeutics Research Group, Institute of Biotechnology, Ferdowsi University of Mashhad, Mashhad, Iran

**Keywords:** Mesenchymal stem cells, mineral trioxide aggregate, osteo/odontogenic differentiation, regenerative medicine

## Abstract

The significance of dental materials in dentin-pulp complex tissue engineering is undeniable. The mechanical properties and bioactivity of mineral trioxide aggregate (MTA) make it a promising biomaterial for future stem cell-based endodontic therapies. There are numerous *in vitro* studies suggesting the low cytotoxicity of MTA towards various types of cells. Moreover, it has been shown that MTA can enhance mesenchymal stem cells' (MSCs) osteo/odontogenic ability. According to the preferred reporting items for systematic reviews and meta-analyses (PRISMA), a literature review was conducted in the Medline, PubMed, and Scopus databases. Among the identified records, the cytotoxicity and osteo/odontoblastic potential of MTA or its extract on stem cells were investigated. Previous studies have discovered the differentiation-inducing potential of MTA on MSCs, providing a background for dentin-pulp complex cell therapies using the MTA, however, animal trials are needed before moving into clinical trials. In conclusion, MTA can be a promising candidate dental biomaterial for futuristic stem cell-based endodontic therapies.

## 1. Introduction

Based on laboratory and clinical studies, mineral trioxide aggregate (MTA) overcame the drawbacks of many conventional substances, and it has been considered as a suitable surrogate in the clinical practice [1–3]. Chemical assessments have shown that in contrast to Portland Cement, MTA contains smaller pieces and fewer harmful heavy metals [4–7]. Hence, it has received much attention as an ideal dental material for various endodontic uses, including pulp capping, pulpotomy, root perforation treatment, and root canal filling [1,8,9]. Several investigators have evaluated the physical properties of MTA, and although physical characteristics can be influenced by methods of placement and environmental conditions, MTA exhibits favorable sealing ability, solubility and compressive strength [10–12]. MTA setting is initiated in the existence of moisture. In the course of hardening, a release of calcium hydroxide and a rise in pH could be detected. Bismuth was reported as another crucial component in this mixture whose solubility increases in acidic conditions, similar to inflammatory environments, and accordingly the precipitation rate of calcium hydroxide in hydrated MTA increases [2,7,13]. Among various MTA types, several studies focused on gray (GMTA) and white (WMTA) forms of this material. The results revealed lower level of hydroxyapatite formation within hydrated WMTA. On the other hand, more silica, calcium, and phosphorus were present in WMTA [13–15].

When in direct contact with phosphate-buffered saline (PBS), a layer resembling the hydroxyapatite structure was produced over MTA-filled root canal walls [16]. Numerous *in vivo* and *in vitro* investigations confirmed good durability and sealing ability of the said layer. Thus, it could be concluded that MTA can develop an appropriate barrier as a filling material in endodontic procedures [3,6,8,16].

Contradictory results on the antimicrobial nature of MTA could be attributed to the type of MTA, the preparation method, and the species of examined microorganism. Some studies indicated that the antibacterial and antifungal effects of MTA were concentration and time-dependent [17–19]. Although some authors believed that it provides a broad-spectrum toxicity against bacteria and fungi, others reported limited antifungal effect by GMTA and no antibacterial impact on strict anaerobes [18,20].

The purpose of this review is to determine what is currently known about the *in vitro* effects of MTA on MSCs, including its cytotoxicity, differentiation-inducing potential, and the signaling pathways through which these effects are mediated. We begin the review with an overview of the current state of knowledge of cytotoxicity of MTA towards MSCs derived from different sources. We continue with an overview of *in vitro* approaches recruited to establish MTA-conditioned media. We then present a summary of studies that have examined MTA’s effects on the expression of osteo/odontoblastic gene markers, mineralization potential of MSCs, and conclude with ideas for future research.

## 2. Material and methods

### 2.1. Inclusion criteria and search strategy

**Figure 1.**
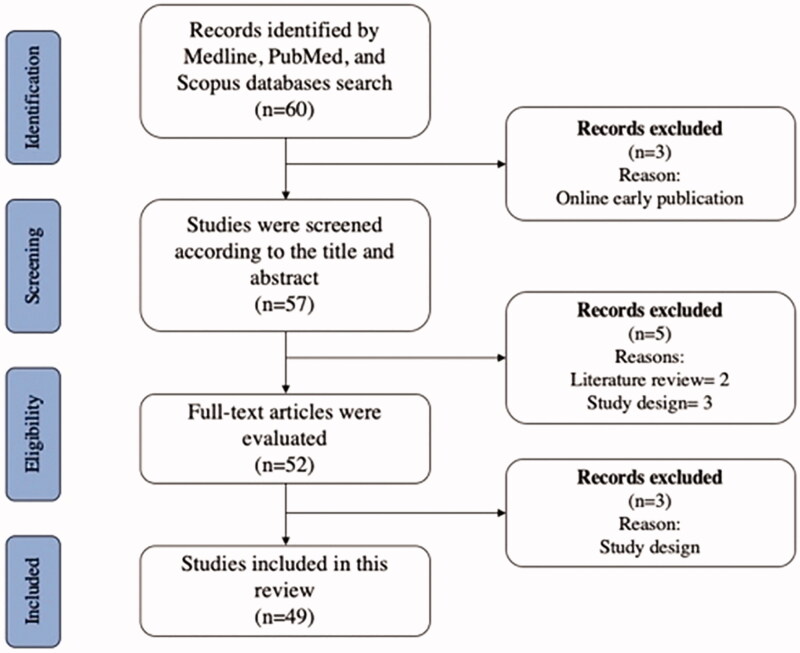
The selection process of the included literature.

In July 2020 a literature review was conducted in the Medline, PubMed, and Scopus databases following the preferred reporting items for systematic reviews and meta-analyses (PRISMA) guidelines. The search keywords were: ‘mineral trioxide aggregate’, ‘stem cell’, and ‘oste/odontoblast differentiation’. Using the Boolean operators ‘AND/OR’ along with the addition of synonyms for the keywords, the final search strategy was: ((‘mineral trioxide aggregate’ OR ‘MTA’) AND (‘stem cell’) AND (‘differentiation’ OR ‘osteogenic’ OR ‘odontogenic’). The inclusion criteria were publications in English within the past 12 years (January 2008 to July 2020), investigating cytotoxicity of MTA and its effects on osteo/odontoblastic potential of MSCs. We excluded review articles, case reports, narratives, and online early published articles. Studies investigating the differentiation-inducing potential of MTA mixture with other root-filling materials, and studies that used solvents other than what was provided by the company were also excluded (Figure 1).

### 2.2. Data extraction and outcomes

Three independent reviewers performed the literature search, title, and abstract scrutinization, and systemic full-text evaluation. Disagreements were resolved by discussion to reach a definitive consensus. Data from each study were extracted by reviewers using an abstraction form, and the following details were collected: Authors, publication year, study type, cell source, MTA preparation protocol, treatment period, differentiation and cytotoxicity assays, and outcomes.

## 3. Results

### 3.1. Cytotoxicity of MTA towards MSCs

When freshly prepared, due to its alkalinity, the cytotoxicity of MTA mixture is high [15,21]. However, Sarkar et al. in 2005 provided another important characteristic about MTA using EDAX to prove that the formed structure was similar to hydroxyapatite crystals, a major mineral element of dentin and enamel, and from these data, they concluded that MTA shows a noncytotoxic nature [16]. A review by Camilleri and Piit Ford in 2006 highlighted greater noncytotoxic nature of MTA compared to traditional substances [22]. Earlier studies focused on various cell types such as gingival and periodontal ligament derived fibroblasts, human osteosarcoma, odontoblasts, dental pulp cells, and bone marrow cells in direct contact with MTA or its extract[23–27]. Recent studies have turned their attention to and concentrated on dentinogenesis and the differentiation-inducing potential of MTA on MSCs (Table 1). It has been shown that MTA is a nontoxic dental material for bone marrow-derived MSCs [28–32,52]. On the other hand, different MTA-based materials had toxic effects on tooth-resident stem cells in a time and concentration-dependent manner [33,34,36,38,39]. The time allowed for establishing an MTA-conditioned medium is another crucial factor in these studies. In particular, since alkalinity decreases over time, longer extraction time may result in lower cytotoxicity [13,34,36,38–40].

**Table 1.** Cytotoxicity of MTA towards MSCs.

Cell Type	Source	Evaluation	Observation	Reference
BMMSC	Human	DNA quantification assay, Enzyme assay	ProRoot MTA and MTA Plus had stimulatory effects on cell proliferation at different dilutions (1:2–1:20), established from 0.1 g/cm^2^/mL extraction medium after 21 days. However, MTA Fillapex showed high toxicity after 1,7, 14, and 21 days at different concentrations.	Costa et al. [28]
BMMSC	Human	AlamarBlue assay	Viability of cells in direct contact with ProRoot MTA was similar to control group after 1, 3, 5, and 7 days of incubation.	D’Anto et al. [29]
BMMSC	Human	MTT assay	No significant difference was observed between cell viability between bone marrow-derived MSCs, cultured in ProRoot MTA-conditioned medium, and control group after 24 h.	Ashraf et al. [30]
BMMSC	Human	MTT assay	Results indicated that cells cocultured with ProRoot MTA and Micro-Mega MTA discs, placed in transwell inserts, had same viability ratio compared with control group on days 1, 3, and 7. Higher cell viability was seen in treated groups on day 14.	Margunato et al. [31]
BMMSC	Rat	MTT assay	MTA was not toxic at different concentrations (0.002, 0.02, 0.2, and 2 mg/mL) after 3 and 5 days.	Wang et al. [32]
DPSC	Human	MTS assay	After 24, 48, and 120 h of incubation in ProRoot MTA conditioned medium, the viability of cells in control group was significantly higher. Moreover, using propylene glycol as mixing liquid did not affect the cytotoxicity of MTA.	Natu et al. [33]
DPSC	Human	MTT assay	DPSC viability in direct contact with ProRoot MTA after 1, 3, and 7 days was statistically lower than control group. It has been shown that on days 1 and 3 MTA, mixed with distilled water, represented lower toxicity in comparison to MTA that was mixed with 10% CaCl_2_, 5% CaCl_2_, and 2.5% Na_2_HPO_4_.	Kulan et al. [34]
DPSC	Human	MTT assay	Three dimensional DPSC culture, established in collagen type I scaffold, on ProRoot MTA showed similar cell viability compared with control group.	Widbiller et al. [35]
DPSC	Human	MTT assay, Enzyme assay, Flow cytometry	Results confirmed that after setting for 24 h, ProRoot MTA discs in transwell inserts were cytotoxic after 3 days. However, using cyclic aging protocol, re-immersing used-MTA discs in deionized water for 4 days in each cycle and placing them in transwell inserts of new culture vessels for 3 days, reduced the toxicity nature of MTA discs on day 3.	Niu et al. [36]
DPSC	Human	XTT assay, Flow cytometry	By using cyclic aging protocol, they concluded that within the 3^rd^ and 4^th^ cycles no difference was observed in MTA Angelus-treated and control groups in terms of cell viability. Furthermore, dilution factor affected the toxicity of the eluent released by the MTA Angelus.	Bortoluzzi et al. [37]
DPSC	Human	MTT assay	Conditioned medium of ProRoot MTA at concentrations of 10 and 20 mg/mL was cytotoxic after 1, 3, and 5 days. However, cell survival was increased after exposure to 0.1, 0.2, 1, and 2 mg/mL ProRoot MTA on day 5.	Zhao et al. [38]
DPSC	Human	MTT assay	Cytotoxicity of 4 types of MTA-conditioned media (ProRoot MTA, RMTA, Nanohybrid MTA, and MTA Angelus) was assessed. Although Nanohybrid MTA exhibited cytotoxicity at all time intervals, all the other three types showed proliferation stimulating potential.	Jaberiansari et al. [39]
DPSC	Human	MTT assay	ProRoot MTA was allowed to set for two time periods, 1 h and 24 h. Cell viability evaluation showed that cytotoxicity in direct contact with MTA was lower in 24-hour group compared with 1-hour and control groups after 7 days.	Agrafioti et al. [40]
DPSC	Rat inflammatory dental pulp	MTT assay, Flow cytometry	ProRoot MTA conditioned medium was made at concentrations of 0.002, 0.02, 0.2, 2, and 20 mg/mL. It was observed that 0.002–0.2 mg/mL MTA-conditioned media showed no cytotoxicity. Cell proliferation was down regulated at concentrations of 2 and 20 mg/mL after 5 days of incubation.	Wang et al. [41]
SCAP	Human	XTT assay, Microscopic examination	ProRoot MTA discs were placed on the bottom surface of the culture vessel for evaluating the toxicity of MTA after 1, 3, and 7 days. It has been shown that after 1 day, cell viability in MTA group was higher than control group. However, no statistical difference was observed after 3 and 7 days.	Peters et al. [42]
SCAP	Human	Coulter counter, Flow cytometry	2 mg/mL ProRoot MTA conditioned medium was not toxic after 1, 3, 5, 7, and 9 days.	Yan et al. [43]
SCAP	Human	MTT assay	Cell coculturing was performed by placing 1 mg of ProRoot MTA in transwell inserts. 24-hour, 48-hour, and 168-hour results indicated that cell viability was not significantly different from control group.	Saberi et al. [44]
SCAP	Human	WST-1 assay	20 mg disc-shaped set ProRoot MTA, allowed to set for 1 or 24 h, was placed in transwell inserts. It was observed that cell proliferation increased in 1-hour and 24-hour groups on day 1 and days 1 and 5 compared with control group, respectively. No statistical difference was observed in term of cytotoxicity of MTA up to 14 days.	Schneider et al. [45]
PDLSC	Human	MTT assay, Microscopic examination	Cytotoxicity and proliferation stimulating potential of various dilutions of MTA Fillapex eluent were determined after 24, 48 and 72 h of cultur. MTA Fillapex exhibited high cytotoxicity at all concentrations and time intervals compared to control group.	Rodriguez-Lozano et al. [46]
PDLSC	Human	MTT assay, Microscopic examination, Flow cytometry	After exposing to different dilutions of Endoseal MTA eluate, produced according to the International Standard ISO 10993-5, for 24, 48 and 72 h, it was revealed that this material was toxic at all concentrations and time intervals compared to control group.	Collado-González et al. [47]
SHED	Human	MTT assay, Flow cytometry	Cell proliferation was stimulated in presence of MTA Angelus eluate, produced according to the International Standard ISO 10993-5, after 2 and 3 days of incubation.	Collado-González et al. [48]
DPSC, PDLSC, BMMSC	Human	MTT assay	Culturing cells in direct contact with ProRoot MTA after 1 day showed no effect on cell proliferation. After 5 days of incubation, cell proliferation was increased in all three cell types.	Chen et al. [49]
TGSC	Human	MTS assay	Cell viability was similar in cells that were in direct contact with ProRoot MTA compared to cells in control group.	Guven et al. [50]
C3H10T1/2	Mouse	XTT assay	Different concentrations of ProRoot MTA conditioned media were produced. viability of cells was not significantly different from control group.	Lee et al. [51]

Cells; BMMSC: bone marrow-derived mesenchymal stem cell, DPSC: dental pulp stem cell, SCAP: stem cell from the apical papilla, PDLSC: periodontal ligament stem cell, SHED: stem cell from exfoliated deciduous tooth, TGSC: tooth germ stem cell, C3H10T1/2: mouse mesenchymal stem cell.

### 3.2. Differentiation-inducing potential of MTA

While cell and molecular signalings involved in the migration of dental pulp progenitor cells during natural reparative dentinogenesis are not fully understood, recent studies focused on strategies mimicking these natural phenomena [53–57]. Similar to well-known hematopoietic stem cell transplantation procedure, it has faced many difficulties in developing regenerative approaches. These challenges include choosing the best cell source, means of differentiation and lineage potency, and the plasticity of the stem cells [58,59]. Well documented results of pulp capping with MTA suggested that it can provide a surface for adhesion of progenitor cells. Moreover, its low-level cytotoxicity, paracrine effects, and potential to induce expression of osteo/odontoblast gene markers in adult tissue-derived stem cells make MTA an applicable chemical for stem cell-based therapies in dentin-pulp regeneration [60–62].

To elucidate the differentiation promoting potential of MTA, previous researchers recruited three main approaches. Treating cells with freshly prepared MTA-conditioned medium was suggested by Hakki et al. [24]. According to this procedure, it was suggested that the bioactive ingredients of MTA are released after incubating the dried MTA in appropriate media for 7 days to produce MTA-conditioned media. The supernatant could be diluted with fresh media to establish different concentrations. Alternatively, cells can be cocultured with MTA discs of diverse sizes, placed in transwell inserts [63]. The third method is to culture the cells in direct contact with MTA. Through this method, one can evaluate the role of MTA in cytodifferentiation, cell migration and adhesion. This shows how this method can be considered to be more comparable to *in vivo* conditions than other methods [64,65].

Evidence from transwell migration assay suggests that MTA can enhance the adhesion and migration of tooth-resident and bone marrow derived MSCs in a concentration-dependent manner [29,45,66]. Additionally, under various conditions, osteo/odontogenic differentiation-inducing potentials of MTA have been reported (Table 2). Based on these studies, MTA can stimulate or enhance the expression of genes involved in upstream and downstream signaling pathways leading to mineralization and production of collagenous and non-collagenous proteins in extracellular matrix. This was confirmed by real-time PCR, Western blotting, and microscopic examinations.

**Table 2.** Differentiation-inducing potential of MTA.

Cell type	Source	Medium	MTA treatment	ALP activity	Mineralization	Evaluated genetic markers	Altered genetic marker(s)^a^	Altered protein marker(s)	Reference
BMMSC	Human	αMEM + Ascorbic acid	MCM	+	+	N	N	N	Costa et al. [28]
BMMSC	Human	DMEM	MCM	M	M	*COL1*, *OCN*	Neither	N	Ashraf et al. [30]
BMMSC	Human	OM	TI	+	+	*COL1A*, *ON*, *RUNX2*	*RUNX2*	M	Margunato et al. [31]
BMMSC	Rat	DMEM	MCM	+	+	S	*ALP*, *RUNX2*, *OSX*, *OCN*, *DSPP*	RUNX2, OSX, OCN, DSP	Wang et al. [32]
DPSC	Human	DMEM	MCM	N	–	S	*ALP*, *OCN*, *RUNX2*, *DSPP*, *MEPE*	N	Natu et al. [33]
DPSC	Human	αMEM + Ascorbic acid	DC (cells on collagen scaffold)	+	N	*COL1*, *ALP*, *DSPP*, *RUNX2*	*COL1A*, *DSPP*	N	Widbiller et al. [35]
							*RUNX2*		
DPSC	Human	OM	TI	–	–	*ALP*, *OCN*, *IBSP*, *DSPP*, *RUNX2*, *DMP1*	*ALP*, *OCN*, *IBSP*, *DSPP*, *DMP1*	N	Bortoluzzi et al. [37]
DPSC	Human	αMEM	MCM	N	N	S	*ALP*, *DSPP*, *COL1*, *OCN*, *IBSP*	N	Zhao et al. [38]
DPSC	Human	DMEM	DC	N	+	*FGF4*/FGF4, *BMP2*/BMP2, *BMP4*/BMP4, *TGF-β1*/TGF-β1, *ALP*, *COL1*, *DSPP*, *DMP1*	*ALP, DSPP, DMP1, TGF-β1*	TGF-β1, FGF4	Asgary et al. [67]
							*BMP4*	BMP4	
DPSC	Human	OM	TI	+	+	S	*ALP, RUNX2, OSX, IBSP, OCN, DMP1, DSPP*	OCN, DMP1, DSPP	Niu et al. [68]
DPSC	Human	OM	MCM	N	+	*OPN, RUNX2, OCN, ALP, COL1*	*OPN, RUNX2, OCN, ALP*	N	Hanafy et al. [69]
DPSC	Human	OM	MCM	+	+	*RUNX2, OCN, COL1*	*RUNX2, OCN*	N	Maher et al. [70]
DPSC	Rat inflammatory dental pulp	αMEM	MCM	+	+	S	*ALP*, *RUNX2*, *OSX*, *OCN*, *DSPP*	RUNX2, OSX, OCN, DSP	Wang et al. [41]
SCAP	Human	αMEM	MCM	+	+	*ALP*, *DSPP*, *RUNX2*/RUNX2, *OSX*, *OCN*/OCN, *IBSP, IL1*α *, IL1β, IL6,* DSP	*ALP, DSPP, RUNX2, OCN, IL1*α *, IL1β, IL6*	DSP, RUNX2, OCN	Yan et al. [43]
SCAP	Human	αMEM	DC (cells were cultured on dentin discs, occluded with MTA)	N	+	*ALP, DSPP, RUNX2, IBSP*	*DSPP, RUNX2, IBSP*	N	Miller et al. [71]
SCAP	Human	DMEM	MCM	+	+	S	*IBSP, OCN, DSPP, OSX, RUNX2, ALP*	N	Saberi et al. [72]
PDLSC	Human	αMEM	MCM	+	+	S	*RUNX2, OCN, OSX, OPN, DMP1, ALP, COL1*	DSP, RUNX2, OCN, OSX, OPN, DMP1, ALP, COL1	Wang et al. [73]
SHED	Human	DMEM	MCM	N	–	N	N	N	Collado-González et al. [48]
SHED	Human	αMEM	MCM	N	N	S	*DMP1*	N	Araújo et al. [66]
TGSC	Human	OM	DC	+	M	S	*DSPP*	N	Guven et al. [50]
C3H10T1/2	Mouse	OM	MCM	+	N	S	*ALP*, *OCN*, *IBSP*	N	Lee et al. [51]

Proteins, and genes; COL1: Collagen type I, OCN: Osteocalcin, ON: Osteonectin, Runx2: Runt-related transcription factor 2, ALP: Alkaline phosphatase, OSX: Transcription factor SP7 also called Osterix, DSPP: Dentin sialophosphoprotein, DSP: Dentin sialoprotein, MEPE: Matrix extracellular phosphoglycoprotein, DMP: Dentin matrix acidic phosphoprotein, FGF: fibroblast growth factor, BMP: bone morphogenetic protein, TGF-β: transforming growth factor β, IBSP: integrin binding sialoprotein. Cells; BMMSC: bone marrow mesenchymal stem cell, DPSC: dental pulp stem cell, SCAP: stem cell from the apical papilla, SHED: stem cell from exfoliated deciduous tooth, TGSC: tooth germ stem cell, C3H10T1/2: mouse mesenchymal stem cell model. Media; DMEM: Dulbecco’s modified Eagle’s medium, αMEM: alpha modified Eagle’s medium, OM: Osteo/odontogenic medium. MTA treatment; MCM: MTA-conditioned medium, TI: transwell insert containing MTA discs, DC: Direct contact with MTA. Assays; (+): significant increase compared to control group, (-): significant decrease compared to control group, M: no significant change has been seen, S: same as elevated gene/protein markers, N: not evaluated.

^a^ regular cells represent upregulation of gene/protein marker expression, shaded cells represent gene/protein marker downregulation

In particular, osteo/odontogenic markers such as Osteocalcin (*OCN*), Osteopontin (*OPN*), Alkaline phosphatase (*ALP*), Bone sialoprotein (*BSP*), and Collagen I (*COLI*) were upregulated in direct contact with MTA or its eluent. The most crucial point to note is that several studies reported increased expression of Runt-related transcription factor 2 (*RUNX2)*, Osterix (*OSX)*, and *DSPP*. The transcription factor *RUNX2* functions as the master regulator of mineralization-related genes at the early stages of mineralized tissue development directly or through *RUNX2*-related signaling pathways [74]. Consequently, downstream regulators such as *OSX* continue to be expressed at later stages and stimulate differentiation into osteo/odontoblast-like cells and expression of bone or tooth-related genes, specially *DSPP* and *OCN*, which are involved in the nucleation phase of dentin calcification and late stages of bone development, respectively [75–77].

**Figure 2.**
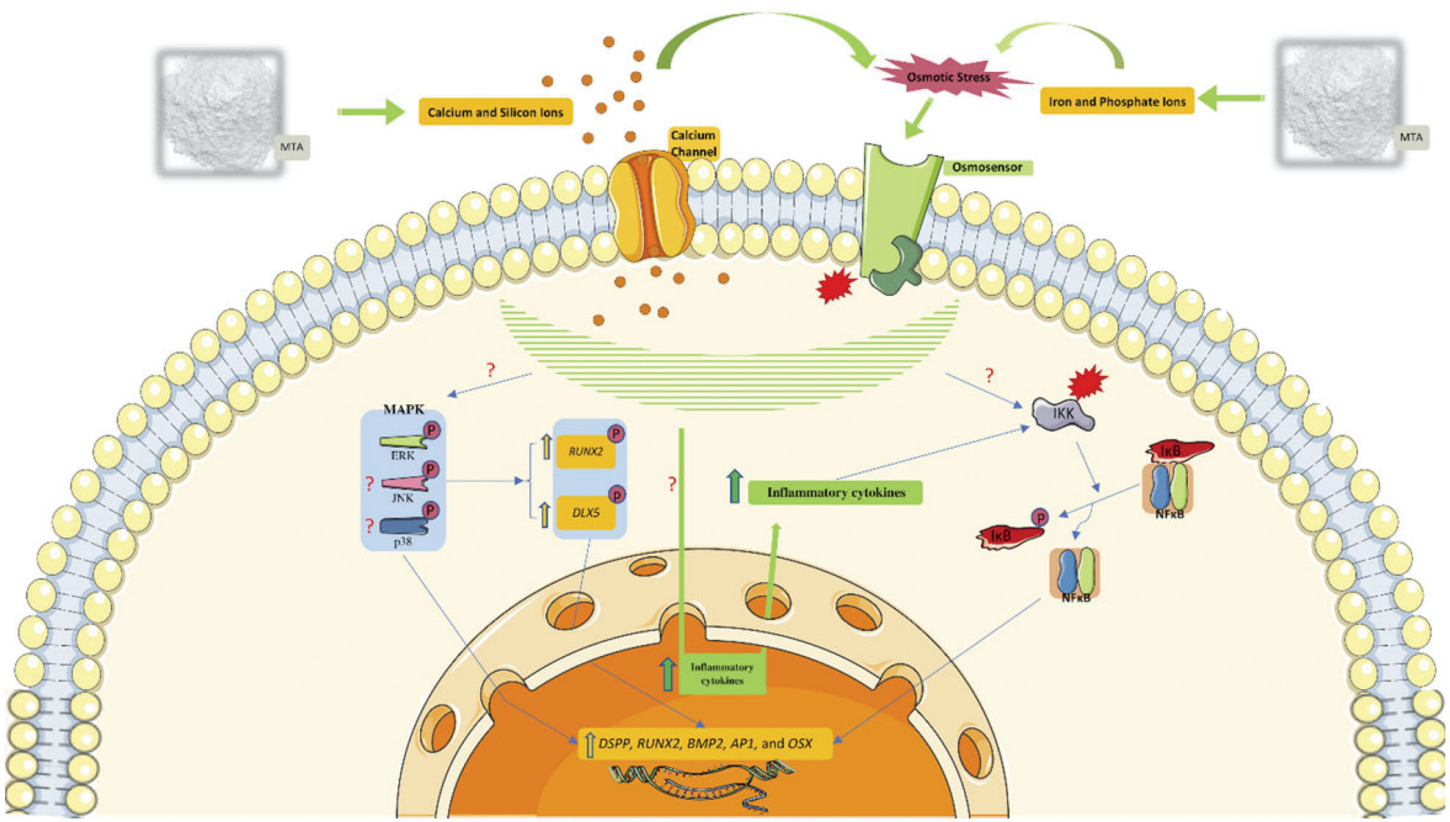
Involvement of major signaling pathways in osteo/odontogenesis effects of MTA on MSCs. IKK: IκB kinase, IκBα masks the nuclear localization signal (NLS) of NF-κB and inhibits its function, AP-1: Activator protein 1, question mark (?) means that the mechanism of action is unknown.

To provide deeper insights into how MTA can promote osteo/odontoblast-like phenotype, signaling pathways that are responsible for hard tissue formation were evaluated in Figure 2. Of the three parallel mechanisms of mitogen-activated protein kinases (MAPKs), i.e. P38, extracellular signal-regulated kinase (ERK1/2), and Jun N-terminal kinase (JNK1/2/3), ERK and P38 have been proved to have a definite role in osteo/odontoblast differentiation. Both routes induce RUNX2 phosphorylation, which increases its transcriptional activity [78–82]. Moreover, P38 has another phosphorylation target, Distal-less homeobox 5 (DLX5), which in phosphorylated form, stimulates *OSX* expression [83–85]. However, the role of JNK in the modulation of hard tissue development is contradictory. On one hand, it is reported that triggered JNK might induce phosphorylation on inhibitory sites of RUNX2, which decreases its transcriptional potential. Contrastingly, it is reported that the JNK pathway can activate osteo/odontogenesis by reducing SMAD6 binding to BMP receptor 1, and making the receptor available for SMAD1 binding [86–88].

Several studies reported that calcium and silicon ions in the MTA-conditioned medium favor ERK and P38 activation by establishing osmotic stress and inward current of these ionic products through calcium channels. This is represented by the reduced osteo/odontogenic-inducing potential of MTA after application of calcium channel blocker and ERK/P38 inhibitors [73,89–91]. On the contrary, results from a few studies were not consistent with the stimulatory influence of MTA on the P38 pathway and suggested that MTA predominantly promotes osteo/odontogenesis through ERK alone or with JNK [32,38]. Furthermore, few studies indicated that the nuclear factor kappa B (NFκB) signaling pathway is intricately involved in osteo/odontogenesis acquired by treatment with MTA. Data from immunofluorescence and Western blot assays confirm that NFκB can be activated after MTA treatment. This might be attributable to the over expression of inflammatory cytokines in response to MTA treatment [41,43,73,92].

Figure 1 was drawn by modifying SMART Servier Medical Art illustrations (http://smart.servier.com/), provided by Les Laboratoires Servier, licensed under a Creative Common Attribution 3.0 Unported License.

## 4. Discussion

Numerous *in/ex vivo* and *in vitro* studies have illustrated extensive applications of MTA in endodontic treatments, specifically including revitalization procedure and stem cell-based dentin-pulp complex therapies. In this review, by organizing the literatures on the modulation of osteo/odontoblast-like differentiation of MSCs through introducing MTA, we came to this inevitable conclusion that various means of MTA treatment can promote mineralization and expression/overexpression of osteo/odontogenic markers in MSCs. However, future studies should continue to minimize experimental shortcomings. For example, they should focus on developing quantitative methodologies and performing experiments with a larger sample size to confirm these conclusions.

*In vitro* evaluations revealed the involvement of major signaling pathways in the effects of MTA on osteo/odontogenesis of MSCs. Unfortunately, these results had some limitations, including limited number of studies and inconsistency of experimental designs. Different types of cell sources and means of MTA treatment have been employed, making it difficult to determine a fundamental mechanism of action. It is worth mentioning that several studies reported cross-talk among different levels of ERK/P38 routes of MAPK and NFκB pathways. Moreover, the activity of these pathways can vary in different organs and cells [93–95]. Accordingly, it is hard to tell whether MTA can trigger simultaneous activation of these routes separately, or it plays a significant role in the stimulation of one of them, which can lead to cross-activation of the other pathways. Future studies should consider these cross-talks and the activity of signaling pathways in different MSCs. Once determined, according to the short- and long-lasting effects of MTA on the specific type of MSCs, a particular protocol including the appropriate MSC’s source and concentration of MTA, can be developed for dentin-pulp complex regeneration when a dental material is involved.

Since the dentin-pulp complex structure could be significantly affected in extensive endodontic lesions, the presence of dental materials in the futuristic stem cell-based endodontic therapies is inevitable. In conclusion MTA could be an appropriate option for this purpose in that not only it exhibits noncytotoxic properties, but also it can induce or upregulate the differentiation of MSCs towards osteo/odontoblast-like cells. However, further studies are necessary to establish to what extent MTA contributes to the regulation of MSCs differentiation.
